# Proteomic Analysis of the Excretory-Secretory Products from Larval Stages of *Ascaris suum* Reveals High Abundance of Glycosyl Hydrolases

**DOI:** 10.1371/journal.pntd.0002467

**Published:** 2013-10-03

**Authors:** Tao Wang, Katleen Van Steendam, Maarten Dhaenens, Johnny Vlaminck, Dieter Deforce, Aaron R. Jex, Robin B. Gasser, Peter Geldhof

**Affiliations:** 1 Department of Virology, Parasitology and Immunology, Faculty of Veterinary Medicine, Ghent University, Ghent, Belgium; 2 Laboratory for Pharmaceutical Biotechnology, Faculty of Pharmaceutical Sciences, Ghent University, Ghent, Belgium; 3 Faculty of Veterinary Science, The University of Melbourne, Melbourne, Victoria, Australia; Khon Kaen University, Thailand

## Abstract

**Background:**

*Ascaris lumbricoides* and *Ascaris suum* are socioeconomically important and widespread parasites of humans and pigs, respectively. The excretory-secretory (ES) molecules produced and presented at the parasite-host interface during the different phases of tissue invasion and migration are likely to play critical roles in the induction and development of protective immune and other host responses.

**Methodology/Principal Findings:**

The aim of this study was to identify the ES proteins of the different larval stages (L3-egg, L3-lung and L4) by LC-MS/MS. In total, 106 different proteins were identified, 20 in L3-egg, 45 in L3-lung stage and 58 in L4. Although most of the proteins identified were stage-specific, 15 were identified in the ES products of at least two stages. Two proteins, i.e. a 14-3-3-like protein and a serpin-like protein, were present in the ES products from the three different larval stages investigated. Interestingly, a comparison of ES products from L4 with those of L3-egg and L3-lung showed an abundance of metabolic enzymes, particularly glycosyl hydrolases. Further study indicated that most of these glycolytic enzymes were transcriptionally upregulated from L4 onwards, with a peak in the adult stage, particularly in intestinal tissue. This was also confirmed by enzymatic assays, showing the highest glycosidase activity in protein extracts from adult worms gut.

**Conclusions/Significance:**

The present proteomic analysis provides important information on the host-parasite interaction and the biology of the migratory stages of *A. suum*. In particular, the high transcriptional upregulation of glycosyl hydrolases from the L4 stage onwards reveals that the degradation of complex carbohydrates forms an essential part of the energy metabolism of this parasite once it establishes in the small intestine.

## Author Summary

The gastro-intestinal nematodes *Ascaris lumbricoides* and *Ascaris suum* are amongst the most prevalent parasites of humans and pigs, respectively. To date, little is known about *A. suum* excretory-secretory proteins, which are present at the parasite-host interface and likely to play a critical role in the induction and development of the immune response. The aim of this study was to identify the excretory-secretory proteins of the migratory stages of *A. suum* utilizing LC-MS/MS. In total, 106 proteins were identified, some of which are known as important players in the parasite-host interface. Interestingly, an abundance of glycosyl hydrolases was observed in the ES material of the intestinal L4 stage larvae. By combining the proteomic analysis with in depth genomic, transcriptomic and enzymatic analyses we could show that the glycosyl hydrolase protein family has undergone a massive expansion in *A. suum* and that most of the glycolytic activity is present in the intestinal tissue of the adult parasites. This could suggest that the degradation of complex carbohydrates forms an essential part of the energy metabolism of this parasite once it establishes in the small intestine. These findings provided useful information on the host-parasite interaction and the biology of this parasite, which can support the concerted efforts to develop better intervention strategies.

## Introduction

Ascariasis is the most prevalent internal macro-parasite of humans (*Ascaris lumbricoides*) and pigs (*Ascaris suum*) worldwide. Approximately 1.2 billion people infected, with a prevalence that is highest in children of the tropics and subtropics [1]. Infected children show signs of malnutrition, growth stunting, intellectual retardation, and cognitive and educational deficits [2]. *Ascaris* also causes major production losses in pigs, including reduced growth rates associated with a decrease in feed conversion efficiency [3]. In addition, lesions in pig livers (i.e. ‘milk spots’) caused by migrating larvae represent considerable losses as such livers are condemned [4]. Traditionally, ascariasis is usually controlled by mass treatment with anthelmintics. However, due to the short activity of the anthelmintics and an environment often highly contaminated with *Ascaris* eggs, reinfections can occur rapidly.

Hosts become infected by the oral ingestion of *Ascaris* eggs containing infective third-stage larvae (L3s). After hatching in the gastrointestinal tract, the larvae penetrate mainly the caecal wall and undergo a hepatopulmonary migration, after which, ultimately, the adult females and males establish and develop in the small intestine. During a primary infection, migrating larvae cause pathological lesions in the gut, liver and lungs. A short-lived immunological reaction against the migrating L3s is seen in the liver 7 days after infection, and is characterized by the production of B cells and CD4^+^ T cells in the local lymph nodes [5]. Two weeks after the infection, the immunological reaction changes from a liver to a lung response, in which the local lymph nodes are enlarged [5]. After the hepatopulmonary migration of the larvae, an intestinal hypersensitivity reaction is seen in the gut, characterized by an accumulation of mast cells, eosinophils and IgA- and IgE-producing cells in the gut mucosa. Pathophysiological changes in the gut, such as increased mucus secretion and mucosal permeability, caused by enhanced secretion of IL-4 and IL-13, have also been observed [6]. After a prolonged exposure, pigs develop a strong protective immunity in the gut, which prevents new incoming larvae from penetrating the intestinal wall. Recently, Masure *et al.* [7] showed that eosinophils play a crucial role in generating this immune barrier.

The proteins produced and presented at the parasite-host interface during these different phases of tissue invasion and migration are inferred to play a critical role in the induction and development of immune responses [8]. Such proteins can be present on the outermost layers of the cuticle and in the excretory-secretory (ES) products, which are mainly released from the cuticular surface, specialized excretory/secretory organs and the worm intestine [8], [9]. To date, little is known about these components from *A. suum*. Limited by technical and practical constraints, earlier studies of ES products from *A. suum* were mainly focused on exploring their chemical composition, ultrastructure and immunological role [10]–[14]. Recently, with major developments in mass spectrometry and genomic technologies, many of the previous challenges and limitations in the proteomic analysis of parasite ES proteins have been overcome, and have led to the characterisation of ES proteomes for parasitic nematodes including *Ancylostoma caninum*, *Brugia malayi, Haemonchus contortus*, *Teladorsagia circumcincta* and *Trichinella spiralis* [15]–[22]. Nonetheless, there has been no profound proteomic analysis of *Ascaris* ES products at critical stages of development. The aim of this study was to characterize the ES proteins of three different larval stages of *A. suum* (i.e. L3-egg, L3-lung and L4) using tandem mass-spectrometry combined with the recently completed *A. suum* genome for annotation [23]. In addition, transcriptomic datasets of the larval stages [23] were used to investigate transcription of genes encoding some of the proteins identified in the ES products from the three larval stages.

## Methods

### Ethics statement

All animal experiments were conducted in accordance with the E.U. Animal Welfare Directives and VICH Guidelines for Good Clinical Practice, and ethical approval to conduct the studies were obtained from the Ethical Committee of the Faculty of Veterinary Medicine at Ghent University (Identification number EC2011/176) who have also approved the document.

### Parasite material

Adult worms of *A. suum* were collected from naturally infected pigs at the local slaughterhouse as part of the normal work at the abattoir. Subsequently, male and female worms were dissected and the intestine, reproductive system and cuticle collected and stored at −80°C until use. Eggs of *A. suum* were obtained from the uteri of female worms, and cultured in 0.1% K_2_Cr_2_O_7_ for 28–30 days at 25°C. After 90% of the eggs had become fully embryonated, the infective L3s were hatched from the eggs as described previously by Urban and Douvres [24] and then separated from eggshell fragments and other debris by baermannization.

Two groups of two pigs were experimentally infected with larvated eggs of *A. suum* by gavage. Pigs of group one were each inoculated with 500,000 eggs and euthanized seven days post infection (pi) in order to collect the lung stage larvae (L3-lung), whereas pigs of group two each received 30,000 eggs and were euthanized 14 days pi to collect intestinal stage larvae (L4). L3-lung and L4 were separated from lung tissue and small intestinal contents of host by baermannization, respectively.

### Preparation and analysis of ES products

All three larval stages (L3-egg, L3-lung and L4) were cultured for five days in RPMI 1640 medium with 10 mM L-Glutamine (GIBCO, Invitrogen) containing 0.2 mg/ml gentamycin (10 mg/ml GIBCO, Invitrogen), 1% amphotericin B (250 µg/ml, Sigma), 1 mg/ml streptomycin (Sigma) and 1,000 U/ml penicillin (Kela pharma). The viability of larvae was checked daily and the culture fluid was collected every 24 h and filtered through a 0.22 µm filter (PALL Corporation). After 5 days, the filtrates were pooled and then concentrated and dialysed against phosphate-buffered saline (PBS) at 4°C using filters (Amicon, YM-10 membranes, Millipore). Proteins were precipitated through the addition of 6 volumes of cold acetone for 18 h at −20°C. The proteins were pelleted by centrifugation at 13,000 rpm for 15 min at 4°C. The pellet was resuspended in PBS and stored in aliquots at −80°C. For SDS-PAGE analysis, protein samples (20 µg per lane) were mixed with loading buffer (2% SDS, 50 mM Tris HCl and 5% β-mercaptoethanol), boiled for 5 min and then separated on 12% SDS-PAGE gels using a standard procedure [25]. After staining with Coomassie Brilliant Blue (Invitrogen), the entire gel lane was sliced in 10 equal pieces (horizontally) and used for subsequent liquid chromatography-tandem mass spectrometric (LC-MS/MS) analysis.

### In-gel and in-solution tryptic digestion and LC-MS/MS analysis

Tryptic in-gel digestion was performed as described previously [26]. In brief, to ensure better transfer of buffers, each protein band was cut into 1 mm^2^ portions, washed twice in 50% acetonitrile with 25 mM ammonium bicarbonate, reduced with 10 mM dithiothreitol in 25 mM ammonium bicarbonate, alkylated with 100 mM iodoacetamide in 25 mM ammonium bicarbonate and digested with trypsin (200 ng per band) at 37°C for 18 h. Peptides were extracted with acetonitrile and dried in a Speedvac.

The in-solution digestion was performed as previously described [27]. In brief, 10 µg of the acetone-precipitated ES proteins were resuspended in 20 µl of 0.5 M triethylammonium bicarbonate buffer, reduced with 2 µl of 10 mM dithiothreitol and incubated at 60°C for 1 h. Subsequently, 1 µl of 200 mM methyl methanethiosulfonate in isopropanol was added and incubated for 10 min at room temperature. The solution was digested with trypsin (resuspended in triethylammonium bicarbonate) in at a ratio of 1/50 (amount trypsin/protein) overnight at 37°C.

Dried peptides were dissolved in 40 µl 0.1% formic acid (FA) and 20 µl was desalted for 10 min on a C-18 pre-column (C18 PepMap100, 5 µm×5 mm, i.d. 300 µm Dionex) with 0.1% FA. Separation was performed by means of reversed phase nano-HPLC (25 cm PepMap C18 analytical column, Dionex) at 60°C using a linear gradient of H_2_O: ACN (97∶3, 0.1% FA) to H_2_O: ACN (20∶80, 0.1% FA) at 300 nl/min over 70 min. The different peptides were analyzed on an ESI Q-TOF Premier (Waters, Wilmslow, UK) in a data dependent mode, with automatic switching between MS and MS/MS for up to 7 higher charge ions, when the intensity of the individual ions rose above 50 counts per sec. Fragmentation of the precursors was performed by means of CID. The capillary voltage was set at 1.9 kV, and the cone voltage was set at 100. M/z ratios for MS ranged between x and y and for MS/MS between x and y. M/z ratios selected for MS/MS were excluded for 150 sec. A custom collision energy profile was used.

### Database searching and sequence analysis

Data were searched against an in-house *Ascaris* sequence database (18,542 protein entries), which is based on the recently published *A. suum* genome [23], using the search engine Mascot Daemon (v.2.3, Matrix Science, London, UK), allowing a maximum of one miscleavage. Carbamidomethyl (C) was specified as fixed modification and carbamidomethyl (N-term), deamidated (NQ) and oxidation (M) were considered as variable modifications for in-gel digest. For in solution digests, methylthio (C) was selected as the fixed modification, and deamidated (NQ) and oxidation (M) as variable modifications. An error-tolerant Mascot search was performed as well. The peptide tolerance and MS/MS tolerance were set to 0.35 Da and 0.45 Da, respectively. Only the most parsimonious group of protein identifications were reported from the identified proteins, and the identification threshold was set at p<0.01. For the proteins that were annotated based on only one peptide, the identification threshold was set at p<0.0001. An estimate of the relative abundance of the predicted proteins in the trypsin digestion was assessed using the Exponentially Modified Protein Abundance Index (emPAI) [28] together with the MS score, sequence coverage, detected peptides numbers. For redundant identifications, the emPAI value from the hit with the highest score was considered. The Gene Ontology (GO) database was used for inferring the molecular function of individual proteins identified. The protein sequences were analysed for the presence of signal peptides and transmembrane regions with SignalP 3.0 and TMHMM 2.0 (http://www.cbs.dtu.dk/services/TMHMM/), respectively. The subcellular localization was predicted with SecretomeP 2.0. The sequences of the identified proteins were then used to BLAST search the *A. suum* genome to identify homologous sequences. This was done through the WormBase (http://www.wormbase.org/) (E-value threshold = 1E-16).

Amino acid sequences of selected eukaryotic glycosyl hydrolases listed in the CAZy database (http://www.cazy.org/) were downloaded and used for multiple alignment and consecutive phylogenetic analyses. These sequences included: *Homo sapiens* alpha acid glycosidase (AAG) (P10253), dual catalytic sucrase-isomaltase (SUIS) (P14410), maltase-glucoamylase (MGA) (O43451), alpha glucosidase AB (GANAB) (Q14697), alpha glucosidase C (GANC) (Q8TET4); *Bos taurus* AAG (Q9MYM4); *Mus musculus* AAG (P70699); *Coturnix japonica* AAG (O73626); *Oryctolagus cuniculus* SUIS (P07768); *Suncus murinus* SUIS (O62653); *Rattus norvegicus* SUIS (P23739); *Sus scrofa* GANAB (P79403); *Drosophila melanogaster* AAG-like (Q7KMM4) and *Caenorhabditis elegans* AAGR1-4. The protein sequences were subjected to MUSCLE alignment (http://www.ebi.ac.uk/Tools/msa/muscle/), and alignments verified and visually checked and edited, as required, in Jalview (http://www.jalview.org/). The program ClustalX 2.0.10 was used to generate phylogenetic tree following analysis using the neighbour-joining method (1000 replicates) [29]. Finally, the program WebLogo application (http://weblogo.threeplusone.com/create.cgi) was used to provide a graphical representation of the amino acid homology around the catalytic sites of some of the glycosyl hydrolases of *A. suum* and *C. elegans*.

### RNA extraction and quantitative real-time PCR (qPCR)

Total RNAs from larvae and adult worm tissue samples were isolated using TRIzol (Invitrogen), followed by further purification with the RNeasy Mini kit (Qiagen), according to the manufacturer's instructions. An on-column DNase digestion was performed using the RNase-free DNase set (Qiagen) to remove any possible genomic DNA. The RNA concentrations were determined (NanoDrop ND-1000 spectrophotometer, NanoDrop Technologies) and its quality was verified (Experion Automated Electrophoresis System, Bio-Rad). For all samples, the RNA quality indicator (RQI) calculated (Experion™ software, Bio-Rad) was 8.0, demonstrating high RNA integrity.

The qPCR analyses were performed as described previously [30]. Tubulin and glyceraldehyde-3-phosphate dehydrogenase (GAPDH) were selected as housekeeping genes. The primer sets used were designed by Primer3 software (http://frodo.wi.mit.edu/primer3/) and are listed in Table S1.

### Analysis of differential transcription

A transcriptome dataset was generated from the L3-egg, the L3-liver, the L3-lung and the intestinal L4 stages as part of a previous study [23]. Briefly, following RNA-seq, all paired-end reads for each library constructed were aligned to the predicted *A. suum* gene set using TopHat. Levels of transcription (reads per kilobase per Million mapped reads (RPKM)) were calculated using Cufflinks [31]. To obtain the RPKM values for genes of interest, accession numbers from the *A. suum* genome were used to search the transcriptomic datasets.

### Protein extraction and enzymatic assays

Protein extracts of larval stages or adult worm tissues were produced by grinding the frozen material to a fine powder in a liquid nitrogen-cooled pestle and mortar. The powder was sequentially subjected to a two-step process with reagents of increasing solubilising power [32]. For the water-soluble protein fraction, 4 ml of PBS, pH 7.4, were used to resuspend the powder for 2 h at 4°C by gentle ‘head-over-head’ mixing. The insoluble material was pelleted by centrifugation at 120,000*×g* for 15 min and the supernatant retained. For the water-insoluble protein fraction, the pellet was incubated at 22°C for 3 h using an extraction buffer consisting of 5 M urea (Sigma), 2 M thiourea (Sigma), 2% CHAPS (Sigma) and 2% SB3-10 (Sigma) in 40 mM Tris, pH 7.4. The supernatant was collected, as described for the water-soluble protein fraction. A general use cocktail of protease inhibitor (Sigma) was added to each extracts to avoid proteolytic degradation. Protein concentrations were measured with the Bradford reagent (Sigma), and proteins stored at −80°C.

The glycosidase assays were conducted by incubating 5 µg of protein extract with 30 mM of substrate at pH 6.5 for 40 min at 37°C. Reactions were quenched by the addition of 3 M Tris. The glucose was quantified using the Glucose Assay Kit (Sigma). The substrates used in the assays included maltose, lactose and sucrose. Each analysis was performed three times, and the results presented as the average of the three readings. For statistical analysis, the unpaired student t-test was used to test differences in activity between the different protein homogenates. The level of significance for analyses was set at P≤0.05.

## Results

### Proteins profiles of the excretory/secretory material

**Figure 1:**
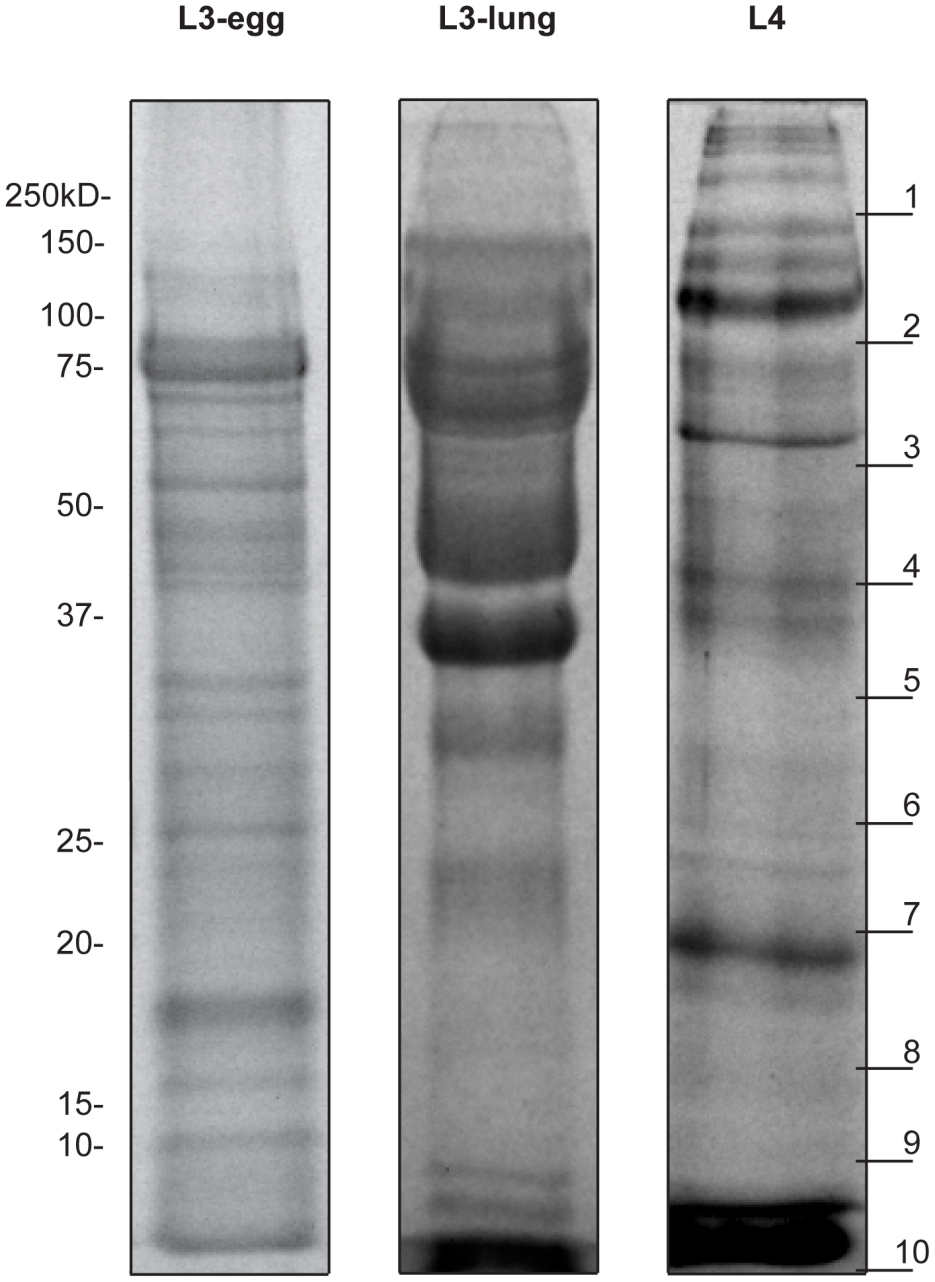
Protein profile of the *A. suum* ES products. Protein profile of the *A. suum* L3-egg, L3-lung and L4 ES products displayed on a 12% SDS-PAGE stained with Coomassie blue. Each lane was loaded with 15 µg of protein. Molecular weight markers are indicated to the left. The 10 gel slices used in the trypsin digests are indicated on the right.

The protein profiles of the ES products from each of the three larval stages of *A. suum*, displayed by SDS-PAGE and Coomassie staining, are shown in Figure 1. The analysis revealed a complex and distinct banding pattern for the ES of three individual stages. Most ES proteins from L3-egg were distributed between 10–120 kDa, whereas those of L3-lung were mainly between 30 and 100 kDa, with a smear above 40 kDa. L4 ES represented a complicated profile, with major bands between 37 and 150 kDa, and some fainter bands in the 20–30 kDa range.

### Protein identifications

**Figure 2:**
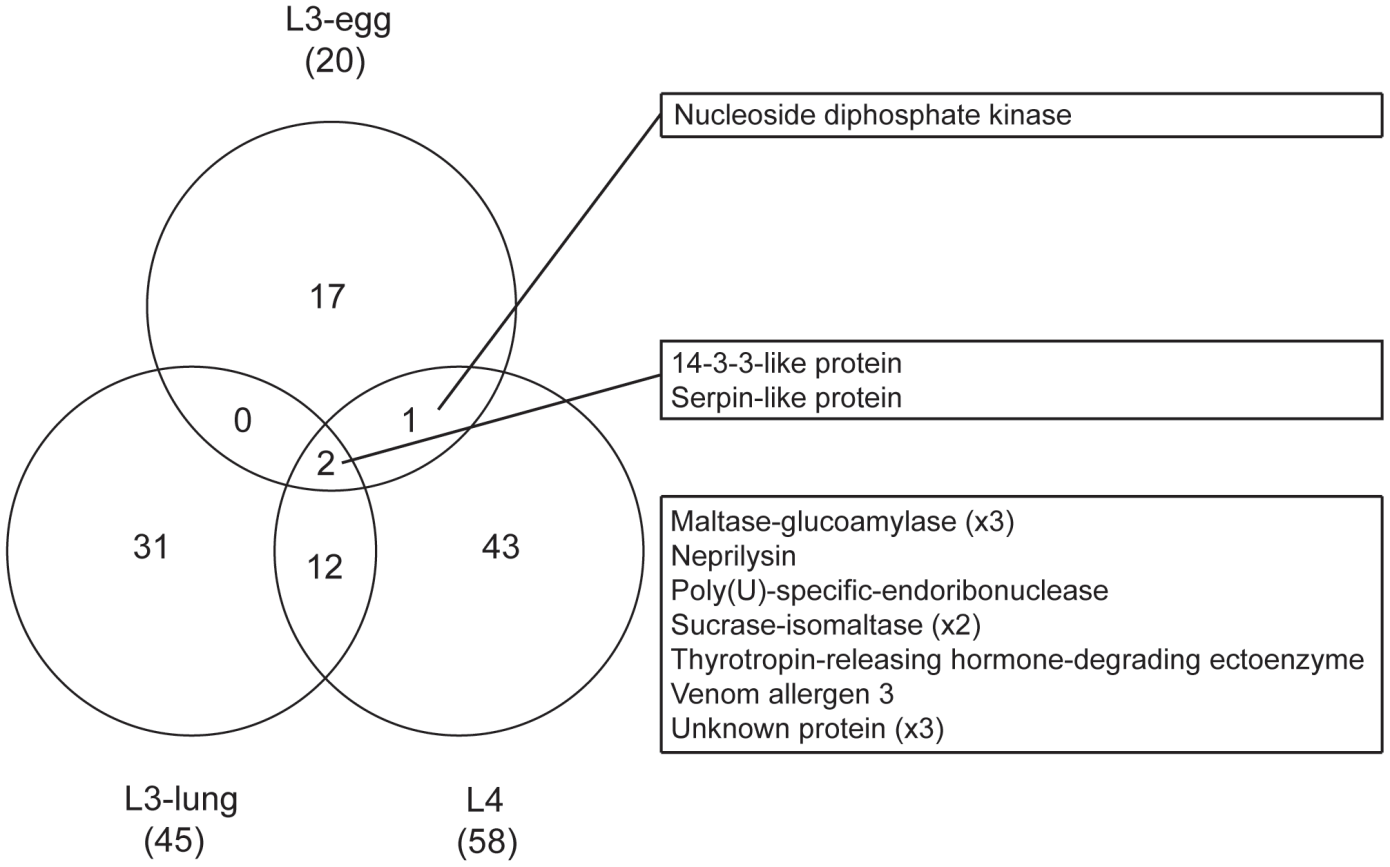
Venn diagram of similar proteins. Venn diagram showing the distribution of the number of proteins identified in ES products from L3-egg, L3-lung and L4 of *A. suum*. The proteins identified are listed on the right.

Mascot searches of the MS/MS spectra for both the in-gel and in-solution approaches yielded 20, 45 and 58 protein identities within ES products of L3-egg, L3-lung and L4 stages, respectively. The full lists of proteins identified are provided in Tables 1 (L3-egg), 2 (L3-lung) and 3 (L4). Most ES proteins detected were inferred to be stage-specific [85% (n = 17) for L3-egg, 69% (n = 31) for L3-lung and 74% (n = 43) for L4], and 15 proteins identified in ES products were shared by at least two larval stages. The identities of proteins shared by all three stages are given in Figure 2. ES products from L3-lung and L4 shared 14 proteins, representing 31% and 24% of their sub-total, respectively, whereas the L3-egg shared only 2 and 3 proteins with L3-lung and L4, respectively. Finally, two proteins shared by all three ES samples included a 14-3-3-like protein and a serpin (Figure 2).

**Table 1:** Protein identifications in *A. suum* L3-egg ES products.

Category/Protein identity^a^	ID^b^	Score	Coverage	Unique sequences	emPAI	Sig^c^
			%			
**Metabolic pathway**						
Endochitinase	GS_04985	205	13	2	0.24	++
	GS_08584/GS_15811^d^	146	18/10	1	0.57	++
**Motor activity**						
Myosin-4	GS_03209	54	1	1	0.02	−
Paramyosin	GS_12985	122	3	3	0.1	−
Tropomyosin	GS_20722	97	6	1	0.12	−
**Binding**						
14-3-3-like protein	GS_05590	174	14	4	0.51	−
32 kDa beta-galactoside-binding lectin lec-3	GS_06140	96	5	1	0.10	+
Globin-like protein	GS_08818	73	12	2	0.21	−
Histone H2A*	GS_13585	99	23	2	0.67	++
	GS_19480	141	25	2	0.67	++
Histone H2B.1/H2B.2	GS_12983	55	19	2	0.67	++
Histone H4	GS_02440	166	40	4	2.3	−
Nucleoside diphosphate kinase	GS_12864	98	6	1	0.33	−
**Other**						
Serpin-like protein	GS_15000	49	19	3	0.27	−
	GS_19115	50	7	2	0.09	−
	GS_19745	77	4	1	0.11	−
**Unknown**						
PAN domain-containing protein	GS_06520	265	12	4	0.27	+
Hypothetical protein CBG_20511	GS_15101	79	11	2	0.08	++
Unknown	GS_06759	125	8	3	0.17	++
Unknown	GS_19262	77	1	1	0.03	−

a The proteins identified were categorized by their molecular function according to information obtained from the Gene Ontology database.

b The accession number in *A. suum* genome database (available on WormBase, www.wormbase.org).

c The identified proteins were predicted to be either a classical secretory protein (+), non-classical secretory protein (++) or not secreted (−) by secretion prediction using SignalP and SecretomeP.

**Table 2:** Protein identifications in *A. suum* L3-lung ES products.

Category/Protein identity^a^	ID^b^	Score	Coverage	Unique sequences	emPAI	Sig^c^
			%			
**Metabolic pathway**						
Maltase-glucoamylase	GS_07553	78	2	1	0.05	+
	GS_15893	142	6	2	0.15	−
	GS_16769	132	8	3	0.18	++
	GS_23879	171	4	4	0.08	++
Neprilysin-1	GS_08219	66	1	1	0.02	+
Sucrase-isomaltase	GS_01568	277	19	4	0.66	−
	GS_02444	258	13	3	0.28	−
	GS_05716	80	11	1	0.19	−
	GS_08447	69	2	1	0.04	++
	GS_17323	129	3	1	0.05	−
	GS_20796	121	2	1	0.04	−
	GS_22047	151	6	3	0.12	++
**Structural**						
Cuticlin-1	GS_10816	63	3	1	0.09	−
Cuticle collagen 12	GS_16238	147	17	2	0.27	++
Cuticle collagen 13	GS_12737	289	8	1	0.34	++
Peptidyl-prolyl cis-trans isomerase B	GS_15602	197	7	2	0.29	−
**Binding**						
14-3-3-like protein	GS_05590	73	3	1	0.11	−
C-type lectin	GS_12842	102	49	2	1.70	−
Latent-transforming growth factor beta-binding protein 1	GS_21305	102	0	1	0.01	++
Thyrotropin-releasing hormone-degrading ectoenzyme	GS_02555	123	4	1	0.08	++
Transmembrane cell adhesion receptor mua-3	GS_11192	253	2	6	0.06	++
**Other**						
Aspartic protease 6	GS_13572	239	9	2	0.25	+
Pepsin inhibitor Dit33	GS_22518	81	6	1	0.13	++
Poly(U)-specific endoribonuclease	GS_22743	101	4	1	0.05	+
Protein DAO-2	GS_24324	120	13	1	0.54	+
Serine protease	GS_07735	78	2	1	0.05	+
Serpin-like protein	GS_19115	303	14	2	0.29	−
**Unknown**						
24 kDa protein of As22	GS_08591	219	17	2	0.36	+
DOMON domain-containing protein	GS_00339	144	20	2	0.43	−
Excretory/secretory mucin MUC-5	GS_22776	529	56	1	4.62	++
Heh-1	GS_20415	66	5	1	0.12	−
Transthyretin-like protein 5	GS_01881	85	9	1	0.25	+
Venom allergen 3	GS_10381	103	12	2	0.26	++
von Willebrand factor domain-containing protein	GS_02090	66	3	1	0.07	++
Hypothetical protein LOAG_00319	GS_14306	72	14	1	0.30	−
Hypothetical protein LOAG_07538	GS_11367	109	5	1	0.12	−
Unknown	GS_01811	72	14	1	0.29	−
Unknown	GS_02698	83	11	1	0.33	−
Unknown	GS_03310	89	4	1	0.14	+
Unknown	GS_09456	119	20	2	0.56	+
Unknown	GS_10718	171	34	2	0.92	+
Unknown	GS_12589	133	11	1	0.58	+
Unknown	GS_15853	245	18	2	0.67	−
Unknown	GS_17230	198	11	2	0.49	−
Unknown	L3E_00366	150	4	1	0.21	−

a The proteins identified were categorized by their molecular function according to information obtained from the Gene Ontology database.

b The accession number in *A. suum* genome database (available on WormBase, www.wormbase.org).

c The identified proteins were predicted to be either a classical secretory protein (+), non-classical secretory protein (++) or not secreted (−) by secretion prediction using SignalP and SecretomeP.

**Table 3:** Protein identifications in *A. suum* L4 ES products.

Category/Protein identity^a^	ID^b^	Score	Coverage %	Unique sequences	emPAI	Sig^c^
**Metabolic pathway**						
Fructose-bisphosphate aldolase 1	GS_19276	189	14	3	0.28	−
Fumarate reductase	GS_20429	78	2	1	0.07	−
Glutathione S-transferase 1	GS_16802	150	11	1	0.16	−
Maltase-glucoamylase	GS_00984	65	5	1	0.13	++
	GS_07553	93	21	1	0.16	+
	GS_15893	642	32	9	1.00	−
	GS_18934	101	7	2	0.20	++
	GS_21210	66	15	1	0.43	++
	GS_23879	1143	16	20	0.46	++
Neprilysin-1	GS_08219	1114	14	14	0.39	+
	GS_10348	198	4	5	0.13	++
	GS_19140	331	6	6	0.14	++
Phosphoenolpyruvate carboxykinase GTP	GS_20378	63	4	1	0.08	−
Sucrase-isomaltase	GS_05716	175	25	3	0.67	−
	GS_08447	97	1	1	0.04	++
	GS_16354	95	5	1	0.08	++
	GS_19777	259	6	4	0.14	++
**Structural**						
Peptidyl-prolyl cis-trans isomerase 3	GS_07454	82	8	1	0.21	++
**Binding**						
14-3-3-like protein	GS_05590	96	3	1	0.11	−
Aminopeptidase N	GS_04166	106	4	3	0.13	++
	GS_05584	143	3	1	0.05	++
	GS_05746	696	14	12	0.29	+
C-type lectin protein 160	GS_02845	194	7	2	0.36	+
	GS_04559	835	35	8	1.76	+
	GS_12996	170	10	3	0.26	++
Enolase	GS_21295	87	3	1	0.08	−
GH family 25 lysozyme 2	GS_22190	441	39	5	3.27	++
Nucleoside diphosphate kinase	GS_12864	66	9	1	0.15	−
Phosphatidylethanolamine-binding protein	GS_22941	103	16	2	0.19	++
Thyrotropin-releasing hormone-degrading ectoenzyme	GS_02555	404	26	7	0.66	++
Zonadhesin	GS_01761	771	22	9	1.00	++
	GS_11354	66	1	1	0.04	+
	GS_11656	693	24	4	0.71	+
**Other**						
Aspartic protease 6	GS_14901	340	15	2	0.17	+
	GS_15316	753	24	5	0.90	+
	GS_19445	919	26	5	1.24	+
Poly(U)-specific endoribonuclease	GS_22743	638	16	7	0.56	+
Serpin-like protein	GS_19115	395	18	3	0.29	−
**Unknown**						
24 kDa protein [Anisakis simplex]	GS_07900	68	16	1	0.41	++
As14	GS_02102	217	25	1	0.26	+
Transthyretin-like protein 5	GS_21838	69	18	1	0.40	−
Transthyretin-like protein 46	GS_02516	119	18	2	0.45	+
Venom allergen 3	GS_10381	131	16	3	0.41	++
Unknown	GS_01911	63	4	1	0.11	++
Unknown	GS_01916	63	6	1	0.20	+
Unknown	GS_01929	89	5	1	0.13	+
Unknown	GS_03310	264	13	2	0.67	+
Unknown	GS_03433	90	3	1	0.08	−
Unknown	GS_04618	125	5	1	0.28	−
Unknown	GS_06231	183	20	3	0.74	+
Unknown	GS_08453	82	11	2	0.30	−
Unknown	GS_08951	613	14	6	0.38	++
Unknown	GS_09456	116	9	1	0.56	+
Unknown	GS_11305	123	1	1	0.03	++
Unknown	GS_23530	126	17	2	0.43	+
Unknown	L3E_00366	124	8	2	0.21	−
Unknown	L4_01560	131	32	2	0.95	++
Unknown	L4_03658	162	20	1	0.31	+

a The proteins identified were categorized by their molecular function according to information obtained from the Gene Ontology database.

b The accession number in *A. suum* genome database (available on WormBase, www.wormbase.org).

c The identified proteins were predicted to be either a classical secretory protein (+), non-classical secretory protein (++) or not secreted (−) by secretion prediction using SignalP and SecretomeP.

*In silico* prediction of classical and non-classical secretion showed that 9 (45%), 25 (56%) and 42 (72%) of the identified proteins from L3-egg, L3-lung and L4 ES products, respectively, were predicted to be either a classical or non-classical secreted protein (Tables 1–3).

**Figure 3:**
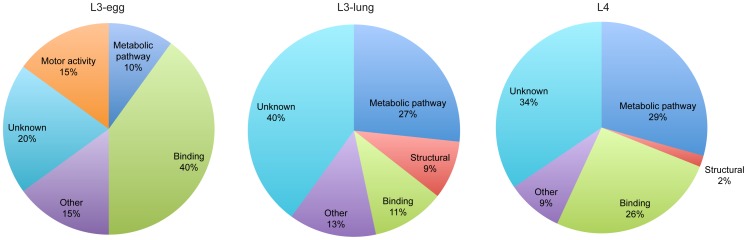
Gene ontology. Gene Ontology terms relating to molecular function assigned to the proteins identified in ES products from L3-egg, L3-lung and L4 of *A. suum*.

All proteins identified were subsequently categorized based on their molecular function, according to information from the GO database. Assigned were: metabolic pathway, structural, motor activity, binding, other functions and proteins of unknown function (Figure 3). From the entire annotated ES protein dataset, 24% (n = 38) of proteins did not have any known function or known homologues in other organisms. Comparison of the results obtained for the three larval stages indicated an increase in the number of proteins involved in metabolic pathways from the L3-egg stage to the L3-lung and L4 stage larvae, whereas only two endochitinase homologues were identified from the L3-egg. In contrast, motor activity proteins, including proteins such as myosin-4, paramyosin and tropomyosin, were unique to L3-egg. Finally, 9% of proteins identified in L3-lung ES products, including cuticlin-1, cuticle collagen 12 and 13, represented ‘structural’ proteins, whereas those belonging to this category were less represented in L3-egg (none) and L4 (2%). Of the 17 binding proteins identified 82% of them were ATP-, ion-, carbohydrate- and DNA-binding proteins.

### Glycosyl hydrolases in *Ascaris suum*

The most frequently identified proteins in ES products were glycosyl hydrolases belonging to family 31 (GH31). In total 16 GH31 proteins were identified in the ES products of L3-lung and L4 larvae with homology to maltase-glucoamylases and sucrase-isomaltases. Six and 5 GH31 proteins were identified in L3-lung and L4, respectively, and another 5 for both of these larval stages.

In order to obtain more information on these proteins, we subsequently BLAST searched the *A. suum* genome for additional members of this GH31 family. In total, 32 protein sequences were identified, all showing homology to GH31 proteins (Table 4). The length of the protein sequences ranged from 80 to 1772 amino acids (aa), suggesting that some of the sequences were not full length. Twenty of the predicted GH31 proteins were predicted as either secreted through a classical or non-classical pathway.

**Table 4:** List of glycosyl hydrolases identified in the *A. suum* genome, their sequence length and their gene levels in different larval stages.

			RPKM values				
Gene ID^a^	AA length	Sig^b^	L3-egg	L3-liver	L3-lung	L4	ES material^c^
GS_05082	735	−	0,2	0,02	0,03	0,02	
GS_16769	587	++	0,06	13,45	0,2	1,14	+
GS_17323	600	+	0,33	141,38	11,02	9,61	+
GS_04731	1047	+	0,02	4,64	1,33	1,05	
GS_10423	226	++	29,27	435,4	182,9	11,19	
GS_00493	153	−	0,11	0,11	0,16	0,09	
GS_01568	311	−	0,05	0,05	0,08	0,05	+
GS_02444	380	−	0,04	0,04	0,06	0,04	+
GS_08026	107	++	0,15	0,15	0,22	0,13	
GS_08447	895	++	0,02	0,02	0,03	0,02	+
GS_16354	408	++	0,04	0,04	0,06	0,03	+
GS_23821	195	+	0,08	0,08	0,12	0,07	
GS_24300	573	++	0,03	1,3	2,52	0,05	
GS_07553	217	+	0,08	0,08	0,11	0,07	+
GS_15893	459	−	0,04	0,04	26,65	0,93	+
GS_22047	830	++	0,02	0,02	0,03	0,02	+
GS_00984	255	−	0,06	0,06	0,09	0,06	+
GS_20796	743	−	0,02	0,02	0,03	0,02	+
GS_21210	80	−	0,2	0,2	0,3	0,18	+
GS_18807	935	−	8,58	17,06	62,88	22,98	
GS_06701	828	+	2,07	2,87	50,98	39,96	
GS_18934	359	++	8,29	2,02	249,19	989,82	+
GS_05716	181	−	0,09	0,09	0,13	1,1	+
GS_13054	457	++	0,22	5,71	15,67	705,7	
GS_17123	737	++	0,02	0,02	0,03	0,48	
GS_21706	521	++	0,03	0,67	0,09	11,18	
GS_23879	1772	++	0,61	0,39	9,98	56,22	+
GS_04250	479	−	1,65	9,45	4,42	38,01	
GS_00096	460	++	0,04	0,11	0,05	97,35	
GS_12078	373	−	0,04	0,04	0,06	3,05	
GS_19777	966	++	1,07	24,87	159,14	1054,46	+
GS_23076	995	+	0,02	0,02	2,49	17,89	

a The accession number in *A. suum* genome database (available on WormBase, www.wormbase.org).

b The identified proteins were predicted to be either a classical secretory protein (+), non-classical secretory protein (++) or not secreted (−) by secretion prediction using SignalP and SecretomeP.

c GH31 proteins identified in the ES material are marked with an ‘+’.

**Figure 4:**
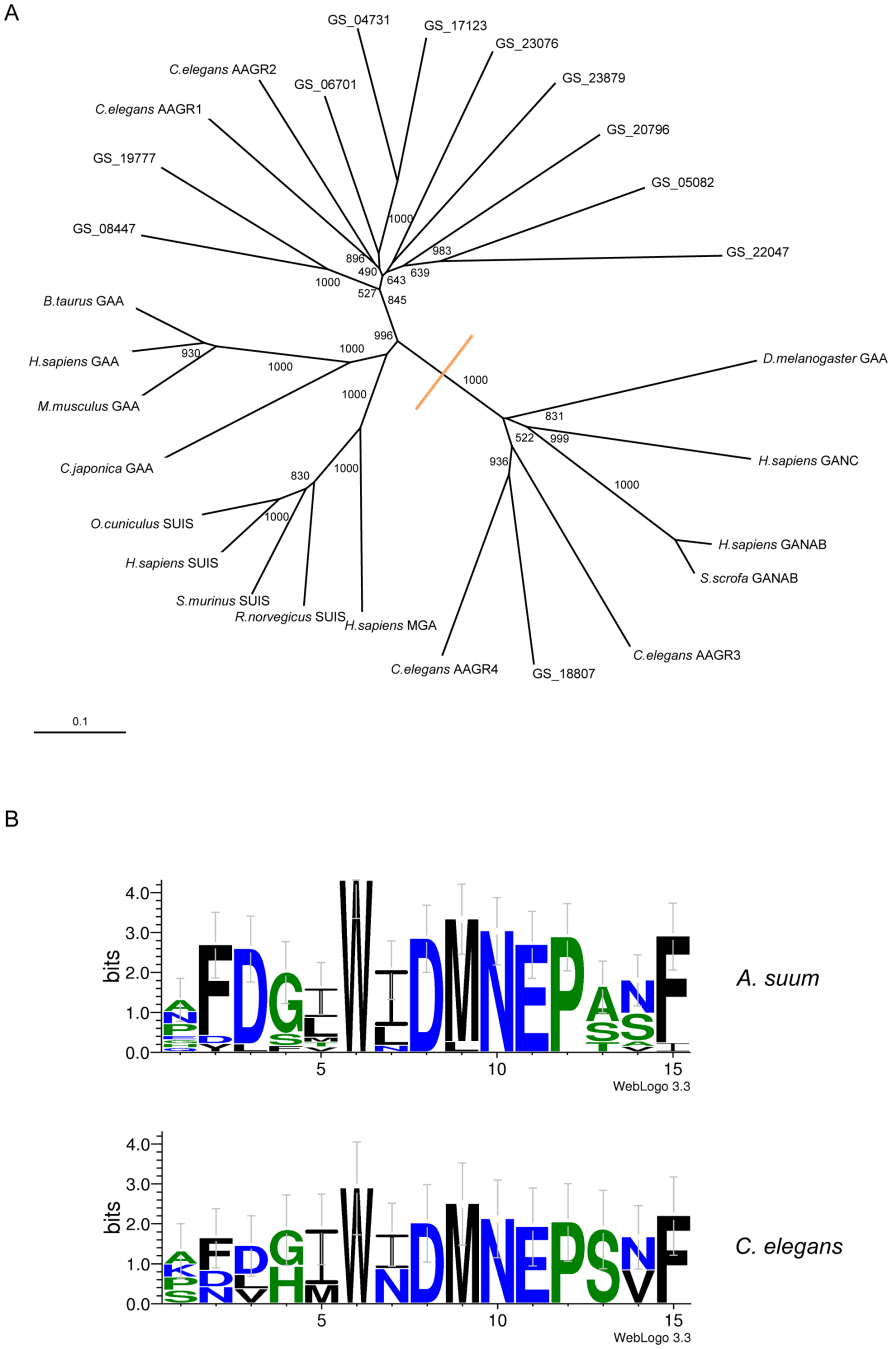
Phylogenetic tree and signature motifs of GH31 proteins. A. Unrooted phylogenetic tree of the *A. suum* GH31 proteins (with a minimum sequence length of 700 amino acids) and other selected eukaryotic GH31 protein following neighbour-joining analysis. The values at the branch nodes represent bootstrap values (maximum 1000). B. Comparative analysis of the amino acids around the catalytic nucleophile (Trp and Asp) of GH31 proteins for the *C. elegans* GH31 proteins AAGR1-4 and the *A. suum* GH31 proteins (GS_04731, GS_05082, GS_06701, GS_08447, GS_13054, GS_17123, GS_17323, GS_18807, GS_19777, GS_20796, GS_21706, GS_22047 and GS_23879).

The GH31 protein sequences (≥700 aa) representing *Ascaris* were aligned with those of homologous proteins from other species for subsequent phylogenetic analysis (Figure 4, panel A). The unrooted tree indicated clustering of the majority of the GH31 proteins of *A. suum* with acid-active GH31 enzymes (i.e. AAG, SUIS, MGA, AAGR1-2), whereas only one (i.e. GS_18807) clustered with neutral-active GH31 enzymes (i.e. GANAB and GANC). The results of a comparative analysis of the amino acid sequence homology around the catalytic site of 13 *A. suum* GH31 proteins (codes GS_0471, GS_05082, GS_06701, GS_08447, GS_13054, GS_17123, GS_17323, GS_18807, GS_19777, GS_20796, GS_22047 and GS_23879) and the 4 GH31 proteins present in *C. elegans* (AAGR1- AAGR 4) (Figure 4, panel B) indicated that the signature motifs around the catalytic nucleophile are largely conserved between these two nematode species.

**Figure 5:**
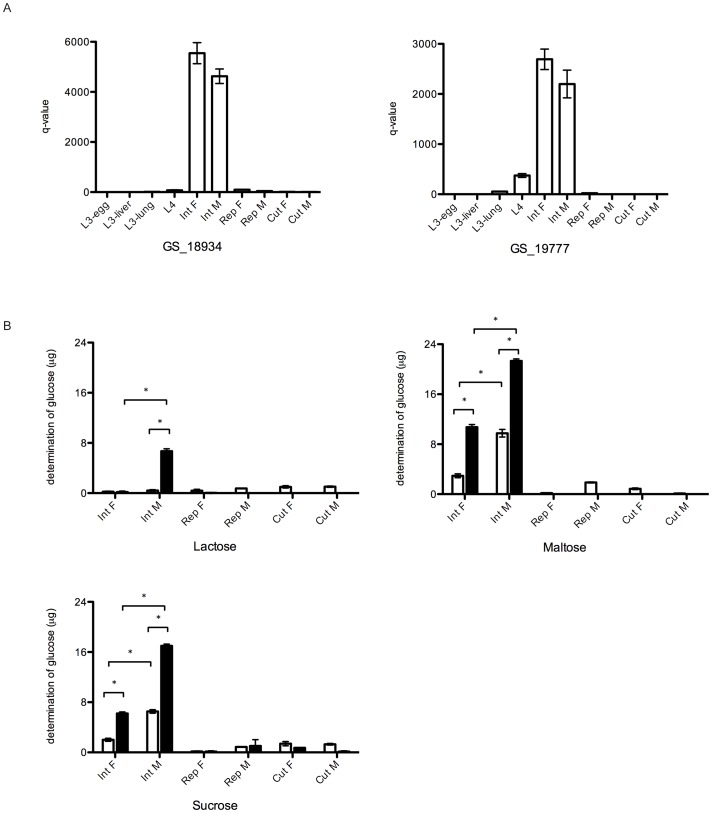
qPCR and glucosidase hydrolytic activity analysis of GH31 proteins. A. A qPCR analysis for two GH31 proteins on cDNA produced from different larval stages and adult worm tissues. B. Comparison of the glucosidase hydrolytic activity in water-soluble (white bars) and water-insoluble extract (black bars) from different adult tissues. Results are shown as average + SD. Substrates used in the assays were lactose, maltose and sucrose. (Int F: female intestine; Int M: male intestine; Rep F: female reproductive system; Rep M: male reproductive system; Cut F: female cuticle; Cut M: male cuticle). (* P<0.05).

In the transcriptomic analysis, the RPKM values for all GH31 proteins identified here showed that most of them are transcriptionally upregulated in the late larval stages (L3-lung and L4) of *A. suum* (Table 4). Based on the RPKM values, GH31 proteins with the highest transcription were GS_18934, GS_13054 and GS_19777, with RPKM values of >500 in L4. A qPCR analysis of genes encoding GH31 proteins (codes GS_18934 and GS_19777) was conducted to (1) verify the transcriptomic data and (2) to analyse their transcription profiles in different tissues of adult *A. suum* (Figure 5 panel A). Indeed, transcription levels of both genes were higher in L4 compared with other stages. In addition, the transcription linked to these GH31 was in the intestine of both female and male adults of *A. suum*, whereas almost no transcription was detected in either the reproductive system or the cuticle of both sexes (Figure 5, panel A).

To confirm the intestinal location of the GH31 proteins, enzymatic assays were performed to measure glycolytic activity in protein homogenates from different adult *A. suum* tissues (Figure 5, panel B). Particularly maltose and sucrose were degraded following incubation with homogenates from the intestinal tracts of both adult male and female worms. The glycolytic activity measured was markedly higher in the water-insoluble protein fractions compared with the water-soluble fraction (P<0.05). In addition, the intestinal homogenates from males showed higher activity compared with females (P<0.05). The degradation of lactose was only observed after incubation with the water-insoluble protein fraction produced from the adult male intestines.

## Discussion

The goal of this study was to identify the ES proteins produced and released by the larval stages of *A. suum in vitro* and to infer the functions of these molecules during the migratory phase of the parasite through the body of the host animal. In total, 106 proteins were identified, of which 62% were predicted to either contain a signal peptide, suggesting secretion through a classical pathway, or predicted to be secreted *via* a non-classical pathway. The other 38% of proteins lacked a detectable signal sequence. Although no changes were observed in the motility or physical appearance of the larvae during the *in vitro* culture, some atypical secreted proteins were detected. The highest number of ‘non-secreted’ proteins for L3-egg was 55% compared with 45% and 26% for L3-lung and L4, respectively. Some of these ‘atypical secreted’ proteins, including 14-3-3 and serpin, may include their secretion in extracellular vesicles as described for other helminths, such as *C. elegans* [33], *Fasciola hepatica* and *Echinostoma caproni* [34]. However, the presence of some typical intracellular proteins in the ES material, such as histones, for example, suggests that there was some cellular damage in the larvae leading to leakage of intracellular proteins into the medium. The precise reason for this is unclear, but it is possible that the hatching procedure, and the subsequent washing steps have a role. Moreover, keeping the *in vitro* culture as short as possible may help reducing the possibly invisible leakage of intracellular proteins into the medium. Therefore, in the future it would be interesting to analyse ES material that has been collected after only few hours of *in vitro* culture.

Among the 106 ES proteins identified in this study, two (i.e. a serpin-like and a 14-3-3 protein) were released by all three larval stages investigated. Serpins are serine protease inhibitors with a wide spectrum of functions in numerous biological systems, such as blood coagulation, complement activation and inflammation [17], [19], [35], [36]. Analysis of the *A. suum* genome and transcriptomes showed that they contain 10 serpin-encoding genes [23], whereas 8 and 3 serpin genes have been identified in the genomes of *C. elegans* and *B. malayi* [35], respectively. A number of studies have previously reported on the presence of serpins in nematode ES products and experimental evidence indicates that many of them can have an immune-evasive function [36]. Interestingly, earlier studies of *A. suum* have shown that the activities of host proteases, such as trypsin and chymotrypsin, were greatly decreased from the micro-environment of live worms with a functioning gastrointestinal system [37]. Subsequently, Martzen *et al.* [38], [39] showed that inactive chymotrypsin complexes were formed in the muscle sarcolemma and in the epithelial surface of the gut of adult *A. suum* as well as in developing eggs and larvae of this nematode. In this way, the serine protease inhibitors may not only protect the worms from degradation in host digestive environment but might also mask the surface of developing larvae, permitting them to evade the host's immune system as they migrate from the intestine to the liver and the lungs. Whether the serpins detected in the ES products from *A. suum* are involved in these processes is still unclear.

In addition to the serpin, a 14-3-3 protein was also detected in the ES material of all three larval stages. Such 14-3-3 proteins represent a family of relatively conserved regulatory proteins, which can bind a range of functionally diverse signaling proteins. In *C. elegans*, a 14-3-3 protein regulates *daf-2*/insulin-like signaling pathway, which is critical for regulating development, longevity, metabolism and stress resistance [40]. Although the 14-3-3 proteins have been isolated and characterized recently as molecules with a significant role in the parasite biology and immunology within the context of the host–parasite relationship [41]–[43], currently, little information is available on their actual role in parasites.

Further comparison of the protein composition of the larval ES proteins showed that more overlap existed between L3-lung and L4 compared with L3-egg. Glycosyl hydrolases belonging to family 31 (GH31) were particularly prominent in ES products from L3-lung and L4. The identification of 16 GH31 proteins is an intriguing outcome of this study, particularly since no other studies have reported the presence of such enzymes in the ES products from nematodes. An analysis of the *A. suum* genome and transcriptomes revealed 32 putative GH31 protein encoding genes/sequences. Although the exact number of GH31 protein genes in *A. suum* is less than 32, because of short or incomplete sequences in the current dataset, it is still clear that this gene family has undergone a large expansion compared with other nematode species. A preliminary analysis indicated the presence of only 4 GH31 protein genes in the genomes of *C. elegans*, *B. malayi* and *T. spiralis* (results not shown). The results presented in the present study also indicated that most of the GH31 proteins were transcriptionally upregulated from the L4 larval stage onwards, with a peak in the adult stage of *Ascaris*, in particular in intestinal tissues. This finding was also confirmed by enzymatic assays, showing the highest glycosidase activity in intestinal protein extracts from adult worms. It has been suggested [44] that *Ascaris* takes most of its nutrients from the partially digested host food in the intestine. The present findings suggest that the degradation of complex carbohydrates forms an essential part of the energy metabolism of this parasite once it is established in the small intestine. The highest level of glycolytic activity was consistently found in the water insoluble protein fraction, suggesting that the enzymes are associated or directly bound to a cell membrane. In mammals, it has been shown that the sucrose-isomaltase complex is anchored to the small intestinal brush border through a highly hydrophobic segment in the N-terminal region of the isomaltase subunit [45], [46]. However, none of the *A. suum* GH31 protein sequences were predicted to contain such a transmembranic region. Therefore, further research is needed to determine the exact cellular location of the GH31 proteins within the intestinal tissues of *A. suum*. If the results would show that they are actually located on the intestinal surface, it would make them interesting drug and/or vaccine targets.

Apart from the shared proteins, most of the proteins identified were unique to a particular larval stage. Amongst the ES proteins identified in L3-egg, there were at least two different endochitinases. Chitinases are enzymes that catalyze the hydrolysis of beta-1, 4-*N*-acetyl-d-glucosamine linkages in chitin polymers. Studies of *B. malayi* (a filarioid nematode) showed that a chitinase was secreted during the exsheathment process of the microfilariae in the mosquito vector and from the eggshell during hatching of the larvae within the reproductive tract of the adult stage [47]. RNAi studies of *Acanthocheilonema viteae* (also a filarioid) showed that chitinase was also critical in the moulting process of the nematode [48]. Interestingly, Geng *et al.* [49] previously reported on the abundant secretion of a chitinase in the perivitelline fluid surrounding the infective *A. suum* larva just prior to hatching from the egg. This chitinase is however different from that identified here. During the larval cultivation, many of the larvae need to lose the L2 cuticle, which is usually still present around the infective L3 larvae when they hatch from the egg. Therefore, it is possible that the chitinases identified herein are involved in the exsheathment process.

Analysis of ES products from L3-lung and L4-ES resulted in the identification of various proteins that have consistently been found in ES material of other parasites, such as the transthyretin-like proteins, C-type lectins and venom allergens [15], [17], [18], [50]. The transthyretin-like proteins are one of the largest conserved nematode-specific protein families of which the function is still largely unclear. Recent data published by Wang *et al.* [51] on TTR-52, one of the 57 transthyretin-like proteins present in *C. elegans* suggest that these proteins act extracellulary to mediate cell-cell interactions.

C-type lectins belong to a type of carbohydrate-binding protein family, known as lectins. These molecules are widely distributed throughout the animal kingdom and have a diverse range of functions, including cell-cell adhesion, immune responses to pathogens and apoptosis [15], [52]. Notably, C-type lectins were also particularly abundant in the secretions from *T. canis* and hookworms [50], [53]. The recent report of the sequence similarity of C-type lectins from *A. suum* to host to dendritic cell receptors suggests that the parasites may utilize lectins to bind to carbohydrate moieties on the surface of host cells to avoid pathogen recognition mechanisms in hosts [54]. The identification of several C-type lectins in the current study could indeed indicate that they play an important, yet undiscovered, role at the parasite-host interface.

Venom allergens belong to the SCP/TAPS protein family and are basically found in every species investigated so far. Despite the fact that the exact function of SCP/TAPS proteins remains unknown, various studies have shown that they are amongst the most abundant proteins expressed and secreted during the transition from the free-living to the parasitic life stages, suggesting an important role in the onset of parasitism [55]. Compared with the number of SCP/TAPS proteins identified in the ES material of some other species [20], [56], [57], it is surprising that only 2 were identified in the current study. However, this finding is concordant with previous evidence from genomic and transcriptomic datasets [23] indicating the presence of only 12 SCP/TAPS-encoding genes in the *A. suum* with relatively limited transcription levels (results not shown).

In conclusion, this study provides the first in-depth characterization of the ES products from the larval stages of *A. suum*, a crucial step in enhancing our knowledge and understanding of the biology of this parasite and its interactions with its mammalian host. The study provides a basis for further molecular investigations aimed at exploring the biological role of the proteins identified and their potential as vaccine and/or therapeutic targets.

## Supporting Information

Table S1 Nucleotide sequences of the primers used in the qRT-PCR assays.
(DOC)
