# Supplementary material for: Proteomic Analysis of the Excretory-Secretory Products from Larval Stages of Ascaris suum Reveals High Abundance of Glycosyl Hydrolases
Source: PLoS Negl Trop Dis. 2013 Oct 3;7(10):e2467. doi: 10.1371/journal.pntd.0002467 (PMC3789772; doi:10.1371/journal.pntd.0002467)
Supplement: Table S1 — Nucleotide sequences of the primers used in the qRT-PCR assays. (DOC) [file pntd.0002467.s001.doc]

Table S1

| Gene ID | Homology | Forward primer  5’ to 3’ | Reverse primer  5’ to 3’ |
| --- | --- | --- | --- |
| GS_18934 | Maltase-glucoamylase | CTTCTTCGAGTTTCCGAACG | GAGAGGAGCGTTGAGGAATG |
| GS_19777 | Sucrase-isomaltase | ACGAGAGAAAGCGGATACCA | GAACTGAAGCCAGCGTTTTC |
| GS_20130 | GAPDH | CGGTTGTATCGACGGACTTT | TGCTGATGTAAGCGATGAGG |
| GS_23993 | Tubulin | CGAGAGGGTTGAAGATGAGC | ATGTTGCTCTCCGCTTCTGT |
